# The Use of Direct Oral Anticoagulants (DOACs) in the Geriatric Population—How to Overcome the Challenges of Geriatric Syndromes

**DOI:** 10.3390/jcm14134396

**Published:** 2025-06-20

**Authors:** Minerva Codruta Badescu, Diana Popescu, Evelina Maria Gosav, Alexandru Dan Costache, Diana Elena Cosău, Adriana Chetran, Ștefania-Teodora Duca, Sandu Cucută, Ionela Lăcrămioara Șerban, Irina Iuliana Costache Enache, Ciprian Rezus

**Affiliations:** 1 Faculty of Medicine, “Grigore T. Popa” University of Medicine and Pharmacy, 700115 Iasi, Romania; minerva.badescu@umfiasi.ro (M.C.B.); evelina.maria.gosav@umfiasi.ro (E.M.G.); dan-alexandru.costache@umfiasi.ro (A.D.C.); e_cosau@yahoo.com (D.E.C.); adriana.ion@umfiasi.ro (A.C.); stefania-teodora.duca@email.umfiasi.ro (Ș.-T.D.); cucuta.sandu@d.umfiasi.ro (S.C.); ionela.serban@umfiasi.ro (I.L.Ș.); irina.costache@umfiasi.ro (I.I.C.E.); ciprian.rezus@umfiasi.ro (C.R.); 2 III Internal Medicine Clinic, “St. Spiridon” County Emergency Clinical Hospital, 700111 Iasi, Romania; 3 Clinical Rehabilitation Hospital, 700661 Iasi, Romania; 4 Cardiology Clinic, “St. Spiridon” County Emergency Clinical Hospital, 700111 Iasi, Romania

**Keywords:** direct oral anticoagulants, geriatric syndromes, risk of falling, swallowing disorders, delayed DOAC intake, polypragmasy

## Abstract

Because the number of elderly people is increasing worldwide, and the prevalence of cardiovascular risk factors and cardiovascular diseases increase with age, in current clinical practice we are faced with a large number of geriatric patients requiring oral anticoagulant treatment. Our review highlights some of the particularities of using direct oral anticoagulants (DOACs) in the geriatric population. We focused on the difficulties of managing DOAC treatment in the presence of geriatric syndromes. We highlighted the practical steps needed to overcome the challenges related to the risk of falling, cognitive impairment, swallowing disorders, and polypragmasy to improve patient care. We provided data to help guide the choice of anticoagulant and dose.

## 1. Introduction

Advances in the diagnosis and treatment of acute and chronic diseases have led to increased life expectancy worldwide. The current high longevity of the population is frequently reported. The number of people in the US aged ≥ 65 was 58 million in 2022, representing 17% of the population, and is projected to increase to 82 million by 2050. This estimated 47% increase will bring the total rate of Americans aged ≥ 65 to 23% [1]. Europe shares a similar population spectrum. In January 2024, the percentage of the European Union population aged ≥ 65 was estimated at 21.6% [2]. In 2024, one in seven Asians was aged ≥ 60, and the region’s elderly population is expected to grow to one in four people over the next 25 years [3]. Africa is striking in contrast, with an exceptionally young population compared to other regions. Elderly people represent only 5.6% of the population. However, with the largest population growth globally, Africa will face problems related to aging in the coming decades, with current estimates forecasting that, by the end of this century, the number of older adults will grow by 15 times. As an overview, one in ten people worldwide were aged ≥ 65 in 2021, and it is expected that, in 2050, one in six people will fall into this age group [4].

Aging is associated with the accumulation of cardiovascular risk factors and implicitly with the increase in the incidence and prevalence of cardiovascular diseases. Atrial fibrillation (AF) is the most prevalent sustained arrhythmia in adults. The lifetime risk of AF is about one in four at age ≥ 40 years. In individuals aged ≥ 55 years, an elevated risk factor profile increases the lifetime risk of AF from one in five to one in three [5]. Over 5-year intervals starting at age 65, the prevalence of AF increased from 6.4% to 10.3%, 15.1%, and 22.4%, respectively, reaching 28.5% at age ≥ 85 [6]. Venous thromboembolism (VTE) also has an incidence that increases with age. The incidence is 3 times higher in patients aged ≥ 65 years than in those of 45–54 years and more than 7–10 times higher in patients aged > 70 years than in those < 55 years [7,8]. Thus, anticoagulants will be necessary at some point in life for many patients, especially older ones.

There are many factors to consider when recommending oral anticoagulant treatment. Some of them are patient-related, such as age, severity of liver or kidney dysfunction, associated comorbidities and their treatment, or the patient’s acceptance/refusal of a particular drug. Other determinants are the indication for treatment—prophylactic or curative, its duration—short or long-term, the characteristics of the drug itself, the route of administration, and the dosage. Direct oral anticoagulants (DOACs) are modern drugs with multiple advantages over classic vitamin K antagonists (VKAs), such as increased efficacy and safety, short onset of effect, predictable pharmacokinetics and pharmacodynamics, few drug interactions, and no need for INR monitoring. Current guidelines provide the necessary recommendations for proper anticoagulation management, generally encouraging the use of DOACs over VKAs [9,10]. Thus, it is expected that the number of DOAC users will continue to increase.

Aging is associated with geriatric conditions, such as multimorbidity, polypharmacy, and geriatric syndromes. The lather includes cognitive syndromes, gait disorders, falls, and frailty. Our review highlights some of the particularities of using direct oral anticoagulants in the geriatric population. We focused on several important aspects related to aging that have an immediate impact on clinical practice, such as the risk of falling, cognitive impairment, and swallowing disorders. We systematized and structured a large amount of data and showed that the problems associated with oral anticoagulation in the elderly are surmountable. We highlighted the practical steps to overcome these challenges and enhance patient care. To our knowledge, our review provides the most up-to-date unified picture of the use of the four DOACs in geriatric syndromes.

## 2. Risk of Falling

Geriatric syndromes include gait disorders and falls. The National Institute for Health and Care Excellence highlights that one in two individuals ≥ 80 years falls at least once a year [11]. Furthermore, 5–10% of all falls in the geriatric population end in severe injuries [12].

Clinicians’ concern about the risk of falls in an elderly person on anticoagulant treatment may lead to the underutilization of this medication. A cohort study evaluating the prescription of oral anticoagulants in elderly patients (age ≥ 75 years) with AF found that age, falls, fractures, major bleeding, and dementia negatively impacted the prescription of these drugs [13]. AF patients aged ≥ 90 years were 40% less likely to receive oral anticoagulants than those aged 75–84. Furthermore, a history of falls reduced the prescription of oral anticoagulants by 17% and a history of fracture by 12%.

Traumatic intracranial hemorrhage (tICH) is most feared. Several studies assessed the risk of tICH after ground-level falls in patients under anticoagulant treatment. One study included 180 patients treated with anticoagulants, with a mean age ± SD of 78.7 ± 12.5 years, of which 31 were on a DOAC [14]. The rate of tICH while on anticoagulant was 1.7%. Of note, no tICH occurred in the DOAC group. Another study included only geriatric patients (age ≥ 65 years) presenting within 72 h of a fall resulting in tICH [15]. Although DOAC-treated patients were more likely to undergo neurosurgical intervention than VKA-treated patients, they had better outcomes, namely, fewer admissions to the intensive care unit, lower 3-day mortality and in-hospital mortality, and fewer discharges to hospice care and inpatient rehabilitation. Strong data are provided by an Italian study that enrolled 234 patients treated with anticoagulants, almost all 65 years or older. Of them, 78 patients were on a DOAC. After mild head injury, tICH occurred in 17% of VKA-treated patients, 5.13% of DOAC-treated patients, and 7.5% of non-anticoagulated controls [16]. Thus, patients treated with a DOAC had a similar prevalence of tICH to that of controls, while patients treated with a VKA had a double prevalence of tICH compared to controls.

After a fall, tICH can be immediate or delayed. Advanced age and treatment with anticoagulants or antiplatelet agents are risk factors for delayed tICH [17]. While immediate tICH is 2.5 times more common in VKA-treated patients than in DOAC-treated patients, the prevalence of delayed tICH is similar between the two classes of oral anticoagulants [18].

A recent systematic review and meta-analysis focused on the incidence of tICH in mild traumatic brain injury patients under oral anticoagulant treatment provides the most accurate data [19]. The analysis included 28 studies, encompassing 5671 DOAC-treated patients and 5501 VKA-treated patients. The overall incidence of tICH in anticoagulated patients was 9.4%. The overall incidence was lower in DOAC-treated patients (7.3%) than in VKA-treated patients (11.3%). The overall incidence of immediate tICH in anticoagulated patients was 8.5%. The incidence of immediate tICH was lower in DOAC-treated patients (6.4%) than in VKA-treated patients (10.5%). The overall incidence of delayed tICH in anticoagulated patients was 1.7%. The incidence of delayed tICH was slightly lower in DOAC-treated patients (1.6%) than in VKA-treated patients (1.9%). Regardless of the type of anticoagulant, there were more immediate than delayed tICHs.

Synthetizing, after mild traumatic brain injury in oral anticoagulated patients, the risk of tICH is generally low, especially in DOAC-treated patients.

In the general population, the benefits of DOACs are undeniable, strongly supported by the results of both pivotal and real-life studies, and highlighted in the recommendations of major guidelines [9,10]. In both AF and VTE patients eligible for a DOAC, they are preferred over warfarin due to better efficacy and safety profiles. It must be emphasized that the risk of ICH is lower with all DOACs compared to VKAs [20]. The decrease is truly significant. Compared to VKAs, the risk of ICH is reduced by 60% with dabigatran, 57% with apixaban, 56% with edoxaban, and 41% with rivaroxaban [20]. Furthermore, in secondary prevention of stroke, DOACs reduce the risk of ICH by 46% compared to VKAs. In a selected population of elderly patients with AF, aged ≥75 years, DOACs were associated with a lower risk of stroke and systemic embolism, hemorrhagic stroke, intracranial bleeding, and fatal bleeding compared to VKAs [21]. A recent meta-analysis in patients with AF at risk of falling found that DOACs are significantly associated with reduced risks of hemorrhagic stroke, major or clinically relevant non-major bleeding, and intracranial bleeding compared to VKAs [22].

However, there has been great interest in identifying the point at which the antithrombotic benefit of anticoagulation is outweighed by the bleeding risk due to falls. An old study estimated that the risk of intracranial bleeding with warfarin outweighs the benefits of treatment only if the patient falls 295 times a year [23]. This means that the patient would need to fall almost every day. Furthermore, the estimates of a 2021 study confirm that, even in patients at risk of falling, the benefit comes from the use of anticoagulants [24]. For the risks to outweigh the benefits, a patient on warfarin would need to fall 35 times a year, a patient on rivaroxaban 45 times a year, and a patient on apixaban 458 times a year.

There are many factors that contribute to an increased risk of falling. A patient’s chronic medication should always be considered and reviewed as it may increase this risk. DOACs are not usually associated with an increased risk of falling. However, some side effects of DOACs should draw attention [25] (Table 1).

Oral anticoagulants that directly inhibit factor Xa seem to be associated with dizziness, hypotension, syncope, and decreased general strength and energy, all significant risk factors for falling. Although the mechanisms involved are still unclear, several possible pathways have been investigated. An experimental study with rivaroxaban was conducted on rat aortic rings [29]. The aortic rings were pre-contracted with phenylephrine. Following the administration of rivaroxaban, a relaxant effect was observed. It was hypothesized that rivaroxaban may have a direct arteriodilatory effect. It was later shown that rivaroxaban can increase nitric oxide synthesis in human arterial fibroblasts [30]. Another laboratory study focused on apixaban [31]. The vasomotor responses of isolated rat mesenteric arteries to vasoconstrictors and vasodilators were assessed in the presence of apixaban. It was shown that factor Xa inhibition induced by apixaban leads to vasodilation mediated by protease-activated receptors (PAR)-2. Further evidence is provided by a study assessing apixaban and rivaroxaban effects on rat aortic rings [32]. It was shown that vasorelaxation was both endothelial cell- and NO-dependent. Thus, it can be assumed that vasorelaxation triggered by direct factor Xa inhibitors may contribute to hypotension and decreased cerebral perfusion, further leading to dizziness and falls.

## 3. Delayed Drug Intake

Although mild forgetfulness is part of the normal aging process, serious memory problems are the expression of a disease, such as cognitive impairment or dementia. Delays in taking a dose or skipping a dose can have significant consequences, especially when it comes to such a sensitive treatment as anticoagulant therapy. Lack of treatment increases the risk of thrombosis, while taking multiple doses increases the risk of bleeding. The short half-life of DOACs is usually regarded as an advantage, especially in the case of bleeding or before surgery. However, a delayed/missed dose causes concern.

**Figure 1:**
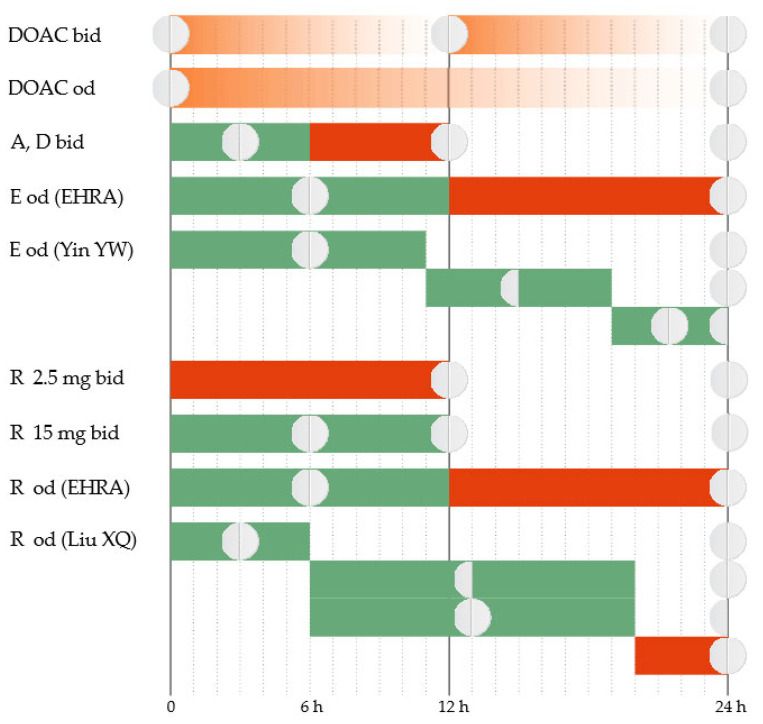
Correct administration of DOAC (DOAC bid and DOAC od). Treatment administration options in case of delay (A = apixaban, D = dabigatran, E = edoxaban, R = rivaroxaban, od = once a day, bid = twice a day). Green = Yes; Red = No.

The total plasma clearance of apixaban is ~3.3 L/h [33], and the elimination half-life is 10–14 h [34]. Apixaban has a twice-daily administration protocol. In the case of a delay in administering the dose, further action depends on the duration of the delay. The regular dose can be taken immediately if the delay is up to 6 h [35]. If the delay exceeds 6 h, the omitted tablet will no longer be taken. The treatment will continue with the regular dose at the next scheduled time (Figure 1).

Edoxaban has an estimated total plasma clearance of 22 L/h [33], and the elimination half-life is 9–11 h [34]. Edoxaban has a once-daily administration protocol. The package insert approved by the Food and Drug Administration (FDA) and the European Medicines Evaluation Agency (EMEA) instructs that, when a dose is omitted, it should be taken as soon as possible on the same day [36,37]. If the day has passed, the omitted tablet will no longer be taken, and the next scheduled dose will be administered as normal. Two tablets should not be taken on the same day to compensate for the skipped one.

Several management features have been highlighted recently for AF patients. The European Heart Rhythm Association (EHRA) emphasizes that there should be a more precise time limit to guide decision-making [35]. It recommends that the omitted edoxaban tablet be taken as soon as possible if the delay is up to 12 h. If the delay exceeds 12 h, the omitted dose will no longer be taken. The treatment will continue with the regular dose at the next scheduled time. Recent evidence suggests that further refinement may be necessary. New data come from simulations based on established population pharmacokinetic (PK)/pharmacodynamic (PD) parameters of the drug [38]. The proposed strategy is to administer immediately the omitted tablet if the delay is up to 11 h. The next day, the treatment will continue as usual. If the delay is 11–19 h, a half dose can be taken, and then the regular dosing schedule can be resumed. If the delay exceeds 19 h, a full dose can be taken and then a half dose at the next scheduled intake, and then the regular dosing schedule can be resumed.

Rivaroxaban has an estimated total plasma clearance of 10 L/h [33], and the elimination half-life is 7–11 h [34]. Depending on the indication, rivaroxaban has a once-daily or twice-daily administration protocol. The 2.5 mg twice-daily regimen is indicated in patients with stable coronary artery disease to reduce the risk of major cardiovascular events and in patients with peripheral artery disease to reduce the risk of major thrombotic vascular events [39,40]. If a 2.5 mg tablet is omitted, the dose will be skipped, and the patient will resume regular treatment at the next scheduled time. The 15 mg twice-daily regimen is indicated for the first 21 days after acute VTE. If a tablet is omitted, it can be taken immediately to ensure a curative daily dose of 30 mg. It is allowed that the two daily 15 mg tablets be taken simultaneously. The 20 mg, 15 mg, or 10 mg once-daily regimes are used in patients with AF to reduce the risk of stroke and systemic embolism or for the prophylaxis and treatment of VTE. If a tablet is omitted, it can be taken immediately on the same day. If the day has passed, the omitted tablet will no longer be taken, and the next scheduled dose will be administered as normal. The dose should not be doubled within the same day to compensate for the omitted tablet. These recommendations of the package insert are approved by both the FDA and the EMEA [40,41].

In patients with AF who have a once-daily administration regimen, the EHRA recommends that the omitted rivaroxaban tablet be taken as soon as possible if the delay is up to 12 h. If the delay exceeds 12 h, the omitted dose should be skipped, and the treatment should continue with a regular dose at the next scheduled time [35]. Recent evidence emphasizes that further refinement can be necessary in this category of patients. Simulations based on established population PK/PD data of rivaroxaban suggest that a delay duration-based approach would be more appropriate [42]. If the delay is up to 6 h, the omitted tablet can be taken immediately, and then the treatment will be continued the next day as usual. If the delay is 6–20 h, the patient has two equivalent options. The first option is to take immediately a half dose, and then resume the regular dose at the next scheduled time. The second option is to take immediately the regular dose and then a half dose at the next scheduled time, and then resume the regular dose at the next scheduled time. If the delay exceeds 20 h, the omitted dose is skipped, and the regular dose will be taken at the next scheduled time.

Dabigatran has an estimated total plasma clearance of 71–144 L/h [33], and the elimination half-life is 14–17 h [34]. Dabigatran usually has a twice-daily administration protocol. The exception is the primary prevention of VTE in orthopedic surgery when it has a once-daily administration protocol. The package insert for the twice-daily administration protocol is approved by both the FDA and the EMEA and is concordant with the EHRA recommendations [35,43,44]. The 6 h delay limit applies as with apixaban. For the once-daily administration protocol, the omitted dose will be taken on the same day. If the day has passed, the omitted dose will be skipped, and the treatment will continue with the regular dose at the next scheduled time.

Elderly patients with memory problems may not only have delays in taking medication but also have doubts about whether they took the dose at the scheduled time or not. They are faced with weighing the bleeding risk (from doubling the dose if they actually took the tablet at the scheduled time) against the thrombotic risk (from skipping a dose if they actually did not take the tablet at the scheduled time). The EHRA offers management solutions for this particular situation [35]. For the twice-daily DOACs, no intervention is recommended. Treatment will be continued with the next scheduled dose. For the once-daily DOACs, the CHA_2_DS_2_-VASc score will guide the decision-making. In patients with high thrombotic risk (a score ≥ 3), a dose can be administered up to 6–8 h after the uncertain intake. In patients with lower scores, no intervention is recommended. Treatment will be continued with the next scheduled dose.

To overcome these problems, family members or caregivers can supervise the administration of treatment. Medication organizers and medication reminder alarm devices are also available to improve treatment adherence. Their use should be encouraged.

## 4. Accidental Overdosage

The PK and PD of apixaban were evaluated in 32 healthy men who received single, sequential, and escalating oral doses of apixaban spaced at least 5 days apart [45]. Total drug exposure and *C*_max_ increase dose-proportional across the 2.5- to 10-mg dose range, which is the approved dose range for clinical use. However, the exposure was less than dose-proportional for the higher doses (i.e., 25 and 50 mg). After doses of ≤10 mg, the return to baseline occurred within 12–24 h, and after doses of 25 and 50 mg, the effects of apixaban extended beyond 24 h. Of note, a prolonged bleeding time in one patient and a positive fecal occult blood test in another patient were the only bleeding events reported, despite the use of single oral doses of apixaban over a 20-fold dose range. Furthermore, several cases of voluntary massive intoxication with apixaban were reported. The ingested doses ranged between 175 and 300 mg [46,47,48,49,50,51]. All cases were managed without bleeding events related to acute overdose.

The first arm of a phase I study enrolled 85 healthy men and evaluated total exposure and *C*_max_ following single escalating doses of edoxaban 10, 30, 60, 90, 120, and 150 mg [52]. Mean *C*_max_ values normalized for body weight and dose increased until 60 mg, after which they declined. At the 10 mg dose, exposure was less than dose-proportional compared to the 30 mg dose. Furthermore, for the 30–150 mg range, exposure was slightly less than dose-proportional for the higher doses. The second arm of this study enrolled 36 healthy men and evaluated total exposure and *C*_max_ following multiple escalating doses of edoxaban. Volunteers were randomized to receive 10 days of one of the three study regimens, namely, 90 mg once daily, 60 mg twice daily, and 120 mg once daily. Drug accumulation occurred with multiple twice-daily dosing but was negligible with the daily doses. Although doses higher than those approved for clinical use have been studied, few subjects had bleeding events, and all were minor (1 gingival bleeding and 10 prolonged bleedings following venipuncture). One case report highlighted that, after a 750 mg overdose of edoxaban, no bleeding event was recorded [53].

The PK and PD of single oral doses of rivaroxaban up to 80 mg were evaluated in healthy young men [54]. Although PK was dose-dependent, less than dose-proportional increases in *C*_max_ and total exposure were observed at doses above 10 mg. Importantly, no evidence of an increased risk of bleeding across studied dosages was found. Another PK/PD study focused on elderly subjects [55]. Forty-eight healthy subjects, aged 60–76 years, were enrolled in a dose-escalation study. They were randomized to receive a single oral dose of 30, 40, or 50 mg of the study drug or placebo. This study found that PK parameters differed less than dose-proportional between the 30 and 40 mg doses and not at all between the 40 and 50 mg doses. This was interpreted as evidence of a ceiling effect in exposure, explained by the poor solubility of rivaroxaban, which may not be completely absorbed at high doses in the gastrointestinal (GI) tract. The few reported bleedings were minor, namely, three events in 12 patients receiving the highest dose (50 mg). Comparative analysis of the results of the two studies showed that PK and PD of rivaroxaban were generally greater in the elderly than in young subjects. However, there was a linear dose-effect correlation, with only a slightly shallower slope in the elderly than in young subjects, especially at lower rivaroxaban plasma concentrations.

Several cases of rivaroxaban overdose have been reported, some of them with massive amounts of drug [56]. Although high doses of rivaroxaban were ingested, between 200 mg and 5000 mg, hemorrhagic complications have been rarely reported and were generally minor. Three case reports included aged patients. A 63-year-old man ingested 1960 mg of rivaroxaban [57]. At 3 h post-ingestion, he received oral activated charcoal and prothrombin complex concentrate. No bleeding occurred. A 71-year-old man ingested 1940 mg of rivaroxaban [58]. No reversal agents or blood products were used. On hospitalization day 4, a small soft-tissue hematoma occurred on the upper back, and it was managed conservatively. The highest amount of rivaroxaban reported to date was 5000 mg [59]. The 64-year-old woman who ingested this amount had significant bleeding and required administration of the reversal agent.

The first arm of a PK/PD study with dabigatran had a single-dose protocol and enrolled 40 healthy men [60]. They were randomized to receive a single oral dose of 10, 30, 100, 200, or 400 mg dabigatran or placebo. *C*_max_ and total exposure increased dose-proportional across the 10 mg to 400 mg dose range. Thus, dabigatran has a linear PK profile even at high doses. The second arm of this study had a multiple-dose protocol and enrolled 40 healthy men, randomized to receive 50, 100, 200, or 400 mg three times daily of dabigatran or placebo. At steady-state, both *C*_max_ and total exposure showed a dose-proportional increase across the 50 mg to 400 mg dose range. Multiple-day administration resulted in drug accumulation in plasma, as *t*_1/2_ of dabigatran is not dose-dependent.

Voluntary ingestion of very high amounts of dabigatran was reported [61,62]. One patient presented 9 h after the ingestion of 11,000 mg dabigatran and another at 2 h after the ingestion of 1500 mg dabigatran [61]. Both cases were managed expectantly, and no bleedings were recorded. Normal renal function and the absence of additional risk factors for bleeding contributed to this outcome. A 68-year-old woman ingested 18,750 mg of dabigatran [62]. She had no overt bleeding 2 h post-ingestion. However, due to the high amount of drug ingested, she underwent gastric lavage and received activated charcoal. Emergency dialysis was considered. Idarucizumab, a specific neutralizing agent for dabigatran, was administered to avoid bleeding associated with dialysis catheter implantation. Since hemostasis parameters normalized afterward and there was no bleeding, hemodialysis was canceled.

Four poison control center-based studies reported overdose cases with DOACs. Stevenson et al. identified four cases of acute voluntary overdose with dabigatran, with doses ranging from 1800 to 3900 mg. There was only one bleeding event, and that one was mild [63]. Conway et al. reported 10 cases of intentional overdose of dabigatran, with major (1 case) and moderate (3 cases) outcomes but no fatalities [64]. Spiller et al. reported 12 cases of rivaroxaban overdose, up to 1200 mg, in suicide attempts [65]. One patient was treated with fresh frozen plasma. There were no bleeding events in this group. Levine et al. identified 52 cases of DOAC overdose (apixaban, dabigatran, and rivaroxaban), of which 7 experienced major bleeding, mostly gastrointestinal [66].

Summarizing, from PK and PD studies, it can be noted that factor Xa inhibitors (apixaban, edoxaban, and rivaroxaban) have a ceiling effect based on the saturation binding of factor Xa sites. This mechanism could underlie the limitation of the risk of bleeding after an acute overdose. This ceiling effect was not identified with the direct thrombin inhibitor (dabigatran). Although elevated serum concentrations of DOACs have been reported after acute overdose, bleeding was rare. It can be assumed that accidental administration of an additional tablet to the daily amount will rarely result in clinically relevant bleeding.

## 5. Swallowing Disorders

Dysphagia is a geriatric syndrome that affects up to one in three elderly people, usually related to stroke, dementia, Alzheimer’s disease, and Parkinson’s disease [67]. Dysphagia can be oropharyngeal, esophageal, or mixed. Patients who have difficulties swallowing solids have esophageal dysphagia, and those experiencing difficulties in swallowing liquids have oropharyngeal dysphagia. Some patients have neurocognitive disorders so severe that they require a feeding tube. Thus, it may become necessary to facilitate the administration of the drug by crushing and administering it with soft food or by crushing and/or dispersing it in water (Table 2).

As with any administration of a crushed drug, it is important to ensure that the entire amount of the product has been given. Firstly, care should be taken during the crushing process to avoid losing tablet fragments. Using a mortar and pestle may be very handy. Secondly, care should be taken that the entire quantity of crushed drug be administered. Therefore, the mortar and pestle, as well as the dosing cup used for actual administration, should be rinsed with a little water, which should also be given.

In current clinical practice, the apixaban tablet is administered with or without food. The effect of food on apixaban absorption is minimal, causing a decrease of less than 20%. Given the favorable benefit–risk profile observed in several clinical trials, this effect was not considered clinically relevant. Crushed apixaban tablets suspended in water had similar bioavailability with regular tablet administration [70]. Crushed apixaban tablets suspended in applesauce resulted in decreased drug exposure, but the effect was small and considered not clinically relevant. Administration of crushed apixaban tablets via a nasogastric tube (NGT) was as feasible as oral administration [71].

Edoxaban 30 mg and 60 mg tablets are administered with or without food. A modest effect of food (a standard high-fat meal) on edoxaban PK was identified. The modest increase observed in both *C*_max_ and total exposure and the reduced absorption rate in the presence of food were not clinically significant [72,73]. A crushed 60 mg tablet, administered orally mixed in apple puree or administered in a water suspension via NGT resulted in total drug exposure similar to a whole tablet [74]. A small study on 12 patients receiving edoxaban through percutaneous endoscopic gastrostomy assessed steady-state plasma drug concentrations and found that all patients were in the therapeutic range [75].

Rivaroxaban 2.5 mg and 10 mg tablets are administered with or without food, as this lower dosage has 80–100% bioavailability regardless of food intake. However, the bioavailability of higher dosage decreases significantly in the absence of food intake. For instance, the bioavailability of rivaroxaban 20 mg tablets is only 66% in a fasted state [76]. The absorption approached completeness when administered with food. Consequently, 15 mg and 20 mg tablets must be taken with food to increase bioavailability to >80%. The type of food (a high-fat or high-carbohydrate meal) did not influence the PK of rivaroxaban [77]. Rivaroxaban 20 mg tablets administered orally with food resulted in similar total exposure and *C*_max_ whether crushed and mixed in applesauce or administered as a whole tablet. When rivaroxaban 20 mg tablets crushed and suspended in water were administered by NGT and followed by a liquid meal, total exposure was unchanged, but *C*_max_ decreased by 18% [76]. In another study, the bioavailability of rivaroxaban 20 mg tablets co-administered with a predefined standardized meal was similar across three types of administration, namely, orally as a whole tablet, orally as a crushed tablet in applesauce suspension, and as a crushed tablet in a water suspension via an NGT [78].

**Figure 2:**
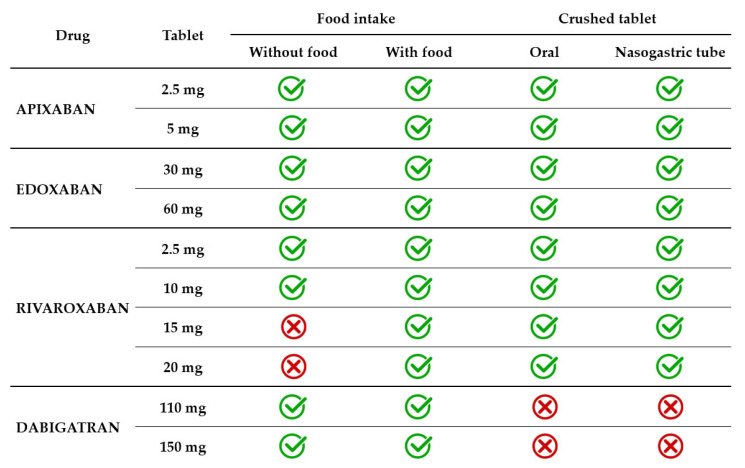
DOAC administration in relation to food and swallowing disorders.

Dabigatran 110 mg and 150 mg capsules are administered with or without food, as food intake does not influence either the total exposure or *C*_max_. It only prolongs by approximately 2 h the time required to reach *C*_max_ [79]. Dabigatran capsules consist of drug-coated pellets inside a shell. The pellets have a tartaric acid core, which creates an acidic microenvironment that increases dabigatran dissolution and absorption [79]. Therefore, unlike other direct oral anticoagulants, dabigatran capsules should not be opened in any way, i.e., by crushing, breaking, or chewing. Tampering with the integrity of the shell will result in significant changes in drug absorption, which will lead to exposure of the patient to an excessive dose. It must be highlighted that the oral bioavailability of dabigatran is 3–7% and increases by 75% after a single dose and 37% after multiple doses if the pellets are ingested without an intact capsule shell [33,44]. Therefore, patients requiring administration of crushed drugs (with swallowing difficulties or enteral tubes) are not eligible for treatment with dabigatran (Figure 2).

## 6. Polypragmasy

Multimorbidity is common in the elderly. The simultaneous use of multiple medications for long-term conditions requires increased attention in prescribing to avoid drug–drug interactions that may increase/decrease the effect of the active substances. The decreased effect of DOACs reduces protection against thromboembolic events, while their increased effect potentiates the risk of bleeding.

Fortunately, DOACs have fewer drug–drug interactions compared to VKAs [80]. However, two vulnerabilities must be considered, one related to DOAC metabolism, namely, the cytochrome P450 (CYP450) enzyme system, and the other associated with DOAC transport, namely, the transporter permeability glycoprotein (P-gp) [81]. Moderate to strong inducers or inhibitors of these pathways may have clinically impactful consequences on circulating DOAC levels. CYP3A4 is an important metabolic pathway for apixaban (20–25%) and rivaroxaban (50%) but not for edoxaban and dabigatran. All four DOACs are substrates of the P-gp, but the variability of drug plasma concentrations is lower for apixaban and edoxaban and higher for rivaroxaban and dabigatran, which could explain the uneven clinical relevance of the drug–drug interactions across DOACs [82]. The 2021 EHRA’s practical guide on the use of non-VKA oral anticoagulants in patients with AF provides reliable and comprehensive guidance to avoid high-risk drug–drug interactions [35]. Among the most commonly used drugs, clinically significant interactions can occur between DOACs and antiarrhythmic, antibiotics, antiviral, fungostatics, anti-cancer, and antiepileptic drugs.

Concomitant administration of antiarrhythmic agents may lead to an increased risk of bleeding [81]. The most suitable options are the combination of amiodarone with apixaban or edoxaban and dronedarone with apixaban, respectively, as dose reduction based on renal function is not necessary. In the remaining scenarios, renal impairment forces dose reduction or compels the patient to avoid association. Among calcium channel blockers, significant interaction is expected with concomitant use of diltiazem or verapamil [81]. Apixaban, edoxaban, and dabigatran can be used safely in combination with diltiazem and apixaban and edoxaban in combination with verapamil. In the remaining scenarios, renal impairment forces dose reduction or compels the patient to avoid association.

A just-published meta-analysis focused on the effects of the association between DOACs and antidepressants indicated that concomitant use of selective serotonin re-uptake inhibitors (SSRIs)/serotonin and noradrenaline re-uptake inhibitors (SNRIs) may increase the incidence of major and intracranial bleeding and recommends caution when using this combination [83]. Anticonvulsants such as carbamazepine, phenobarbital, and phenytoin are strong inducers of CYP3A4 and P-gp. They decrease the plasma level of DOAC and negatively impact the protective effect against thrombosis [81]. Levetiracetam and valproate-containing drugs also decrease the circulating levels of DOAC but through an unknown mechanism. The 2021 EHRA guideline issued a cautionary warning and even banned several associations between DOAC and anticonvulsants [35]. Concomitant administration of dabigatran with carbamazepine or phenytoin, of rivaroxaban with carbamazepine or phenobarbital or phenytoin, and of all DOACs with valproic acid are contraindicated (Table 3).

The combination of anticoagulants and antiplatelet agents carries an increased risk of bleeding. It is used in patients with an indication for long-term oral anticoagulant treatment, such as AF, undergoing percutaneous coronary intervention (PCI). To enhance safety, a DOAC is preferred over VKA and clopidogrel among P2Y_12_-receptor inhibitor antiplatelet agents (P2Y_12_i) within the association. Triple antithrombotic therapy (oral anticoagulant, P2Y_12_i, and aspirin) is indicated after PCI usually for 1 week or up to 1 month in patients with high ischemic risk [9]. Treatment continues with dual antithrombotic therapy (an oral anticoagulant and a P2Y_12_i) for up to 6 months in patients with CCS and up to 12 months in patients with ACS. Subsequently, only the anticoagulant is continued long-term. In patients where concerns about bleeding risk prevail over those about thrombotic risk, a reduced dose of rivaroxaban or dabigatran may be used instead of a full dose. To lower the bleeding risk, correction of modifiable risk factors is beneficial. Alternative analgesics will be offered instead of nonsteroidal anti-inflammatory drugs, and a proton pump inhibitor will be prescribed in patients at high risk of GI bleeding. When assessing drug–drug interactions between DOACs and antiplatelet agents, the only significant interaction found was between ticagrelor and dabigatran [82]. Ticagrelor significantly increased drug exposure for dabigatran. Because ticagrelor is a P-gp inhibitor, it has the potential to increase drug exposure for other DOACs as well.

The use of VKAs in diabetics treated with oral hypoglycemic drugs, particularly sulfonylureas, was associated with episodes of hypoglycemia [84]. In addition to the alleged drug–drug interaction, vitamin K antagonism itself appeared to be a contributor. Vitamin K was shown to have beneficial effects on insulin sensitivity and glycemic control [85]. Thus, by antagonizing vitamin K function, VKAs may interfere with glycemic control and promote hypoglycemia. Free of these shortcomings, DOACs have a significantly lower risk of severe hypoglycemia than VKAs when given concomitantly with antidiabetic drugs [86].

Unrecognized drug–drug interactions are a significant underlying cause for incorrect DOAC administration or dosing, leading to an increased risk of major thromboembolic and hemorrhagic events. Therefore, drug–drug interactions should be looked for at the time of prescribing because dose reduction or avoidance of the combination might be appropriate.

## 7. The Choice of Anticoagulant

In general, DOACs are preferred over VKAs in patients with AF and VTE who are anticoagulant naïve [9,10]. DOACs have demonstrated comparable or superior efficacy and safety to VKAs in patients with AF [87] and comparable efficacy, along with a significantly lower risk of bleeding, to VKAs in patients with VTE [88].

**Figure 3:**
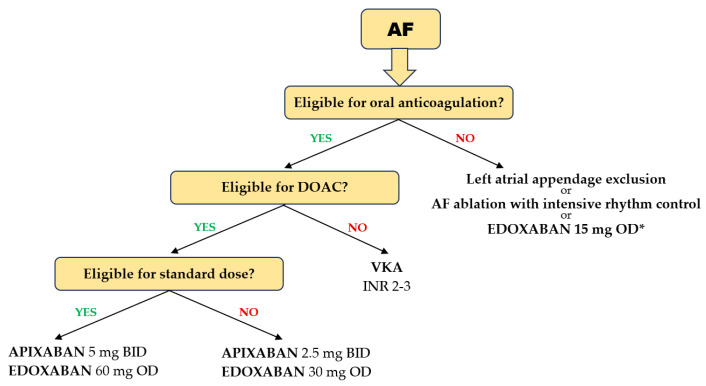
Choice of anticoagulant in elderly and frail patients with AF. (* based on the results of the ELDERCARE AF trial).

Several particularities of DOAC use in the geriatric population should be highlighted. In patients with AF aged ≥ 75 years, pivotal trials showed that each of the four DOACs was more effective in preventing stroke and systemic embolism compared with warfarin, with no heterogeneity between studies. A significant 30% reduction in the risk of ischemic events was achieved with DOACs compared with VKAs [89]. In the same elderly population, the risk of major bleeding was reduced with apixaban and edoxaban compared with VKAs, but not with dabigatran and rivaroxaban, which showed neutral results compared with VKAs [89] (Figure 3). It should be emphasized that a significant reduction in intracranial bleeding was obtained with any DOAC compared with VKAs (>50% reduction with dabigatran, apixaban, and edoxaban and 20% with rivaroxaban) [90]. It is noteworthy that, in the four pivotal studies of DOACs, only 20% of patients were >80 years of age and only 5% were >85 years of age. However, the results were consistent with those in patients > 75 years of age. In very frail elderly patients with AF in the ARISTOPHANES trial, apixaban reduced major bleeding more than warfarin, dabigatran, or rivaroxaban [91]. In addition, dabigatran and rivaroxaban were associated with an increased risk of GI bleeding in patients aged > 75 years, but not apixaban and edoxaban [92]. Another analysis confirmed these results, highlighting that apixaban and edoxaban had the most favorable safety profiles for major GI bleeding. A 44% reduction in major GI bleeding was observed with apixaban compared with rivaroxaban [93].

**Figure 4:**
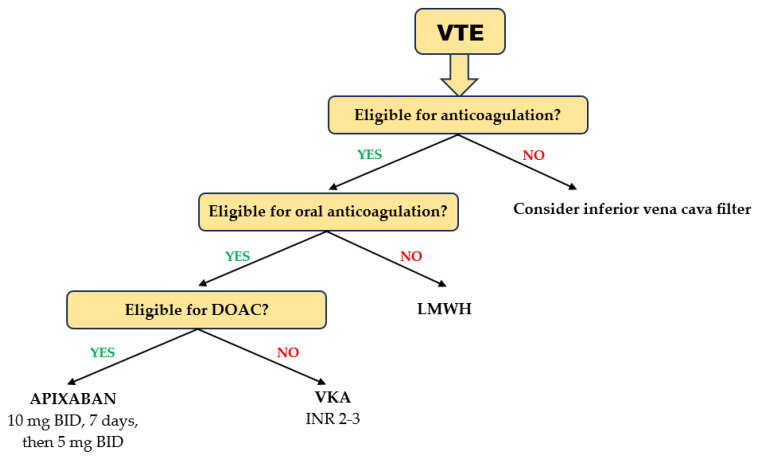
Choice of anticoagulant in elderly and frail patients with acute VTE.

Patients aged ≥ 75 years treated with DOACs for acute VTE had a lower risk of recurrent VTE and major bleeding than those on VKAs [94]. Rivaroxaban, apixaban, and edoxaban were equally effective, but edoxaban showed a higher risk for clinically relevant bleeding than rivaroxaban and apixaban [95]. Consistent with the results obtained in geriatric patients with AF, a meta-analysis evaluating the efficacy and safety of DOACs in patients with acute VTE aged ≥ 75 years showed that apixaban ranked first in terms of both effectiveness and safety [96] (Figure 4). In elderly patients (age ≥ 75 years) with cancer receiving chemotherapy, the differences in efficacy and safety between DOACs and VKA were little and clinically irrelevant [97]. However, in those without metastasis and in those with higher-risk cancer types, DOACs may be effective in reducing VTE occurrence requiring hospitalization. Elderly patients are at higher risk for major bleeding requiring hospitalization, except those with metastases or at high risk of VTE.

A VKA is preferred over a DOAC in several clinical scenarios. Patients with mechanical prosthetic heart valves with or without AF, those with AF and concomitant moderate-to-severe mitral valve stenosis, and patients with thrombotic antiphospholipid syndrome are not eligible for a DOAC because, in these settings, DOACs showed inferiority to VKAs in protection against thromboembolic events [98,99,100]. There was also evidence of an increased risk of bleeding [98]. Renal impairment (especially for dabigatran) and liver impairment (especially for rivaroxaban) also restrict DOAC therapy [9,101]. While age-related decline in renal function is common and warrants dose reduction or discontinuation of a DOAC, age-related decline in hepatic function is unlikely to be clinically relevant.

VKA treatment is burdened by the narrow therapeutic window, multiple drug and food interactions, and the need for frequent INR monitoring and dose adjustment. Patients with low time in the therapeutic range (TTR < 65%) have unfavorable outcomes, including higher mortality rates [102]. Supratherapeutic levels of VKA increase the bleeding risk, and subtherapeutic levels increase the thromboembolic risk. To optimize the outcomes of these patients, two strategies are available, namely, improving the TTR to ≥70% or switching to a DOAC.

In VKA users, improvement in the TTR can be achieved by increasing treatment adherence, especially by attending scheduled INR check-ups and addressing diet inconsistencies. A TTR-INR-guided VKA adjustment protocol performed well in patients requiring optimization of the TTR [103].

The transition from a VKA to a DOAC is recommended in patients who fail to maintain a TTR ≥ 70% (ESC, class I recommendation) and is justified when there are concerns about intracranial bleeding or reasons related to patient choice following shared decision-making [9,104]. Although there are concerns that a patient who is non-adherent to VKA will not adhere to DOACs either, real-life data show that most patients achieved adequate adherence to DOACs despite a low pre-switch TTR [105]. Recently published results of the FRAIL-AF Randomized Controlled Trial highlighted that switching from a VKA to a DOAC in older (age ≥ 75 years) and frail AF patients resulted in a higher risk of major or clinically relevant non-major bleeding, without an associated reduction in thromboembolic complications [106]. Thus, this study concluded that, in such patients, if the TTR is good, VKA treatment may be maintained instead of switching to a DOAC [9]. In patients with a low TTR, switching to a DOAC can certainly be considered appropriate.

There is high variability in physiological and functional reserve among individuals of the same age because biological age may be in discrepancy with chronological age. Therefore, in elderly individuals, a comprehensive geriatric assessment is recommended to identify frailty and assess its severity. Elderly patients who are severely frail/very severely frail (completely dependent on personal care) and those who are terminally ill (approaching the end of life) may receive limited or no benefit from oral anticoagulant treatment [35]. Therefore, an individualized, patient-centered approach is recommended. The EHRA guideline highlights the utility of the Canadian Study of Health and Aging (CHSA) Clinical Frailty Scale to guide decision-making.

Last but not least, socio-economic conditions can influence therapeutic conduct, as patient out-of-pocket (OOP) costs may lead to discontinuation of DOAC treatment. In a large cohort of US patients receiving anticoagulant treatment for AF or VTE, OOP costs greater than or equal to USD 100 were associated with the highest risk of abandoning the treatment with a DOAC [107]. A meta-analysis assessing the cost-effectiveness of the two classes of oral anticoagulants showed that the net benefits favor DOACs in high-income countries using a third-party payer perspective, while no DOACs were more cost-effective in upper-middle income countries [108]. Thus, local health policies may influence the prescription of anticoagulants.

## 8. The Choice of Anticoagulant Dose

Adequate dosing of DOACs ensures benefits are maintained at the lowest bleeding risk. The doses for DOACs included in AF [9] and VTE [10] guidelines come from RCT results. In patients with acute VTE, only edoxaban has dose reduction criteria, which are based solely on renal function. In patients with AF, all four DOACs have criteria for dose reduction (Table 4). The criteria vary with the DOAC. Age is considered for dose reduction only for apixaban and dabigatran. It is worth noting that renal function is taken into account for all DOACs when choosing the dose. As the elderly usually have renal impairment, a reduced dose is often appropriate. The off-label dose reduction in DOACs in patients eligible for standard-dose anticoagulants is discouraged because of the increased risk of ischemic events [109].

There is a bidirectional interplay between DOACs and renal function. On the one hand, DOACs have variable renal clearance with rates of 80% for dabigatran, 50% for edoxaban, 35% for rivaroxaban, and 27% for apixaban. It should be emphasized that, when choosing between full and reduced dosing, renal function assessed by creatinine clearance is considered; as in all pivotal trials of DOACs, estimated creatinine clearance (eCrCl) calculated with the Cockcroft–Gault formula was used as the threshold for drug dosing. Regardless of the calculation method, i.e., MDRD or CKD-EPI equations, the estimated glomerular filtration rate (eGFR) cannot be used interchangeably with eCrCl, as clinically significant discordances in DOAC dosing may occur. The eGFR may overestimate renal function and assign the patient the full dose instead of the reduced dose, thereby increasing the risk of bleeding events. This is particularly relevant for DOACs with a greater dependence on renal clearance and among elderly patients, where the discordance in dose has been up to 30% [110]. Due to its lower dependence on renal function among the DOACs, apixaban appears to be the most appropriate choice in patients with severe renal impairment. A systematic review concluded that apixaban has similar efficacy and a somewhat superior safety profile to warfarin in patients with stage 4 or 5 chronic kidney disease (CKD) and receiving dialysis [111]. On the other hand, long-term use of warfarin has been associated with worsening renal function. Some DOACs have resulted in better renal outcomes than warfarin. Rivaroxaban has been associated with lower risks of ≥30% decline in the eGFR, doubling of serum creatinine, and acute kidney injury (AKI). Dabigatran has been associated with lower risks of ≥30% decline in the eGFR and AKI. Apixaban has not shown any relationship with renal outcomes [112].

Patients with AF and a total body weight ≤ 60 kg are candidates for a reduced dose of apixaban and edoxaban. In general, lean body mass and total body mass are closely correlated, but aging leads to changes in body composition, namely, a decrease in lean body mass due to decreased muscle mass and water content, and an increase in adiposity. DOACs are hydrophilic drugs; therefore, a decrease in lean body mass leads to a decrease in the volume of distribution and, consequently, to an increase in plasma drug levels, with the potential to augment the risk of bleeding. Although current guidelines recommend dose reduction based on total body weight, recent studies have shown that 16% and 25% of elderly sarcopenic patients had supratherapeutic plasma anti-Xa levels at trough and peak, respectively [113]. Currently, there is an unmet need for guidance on DOAC dose adjustment in elderly sarcopenic patients.

The ELDERCARE AF trial showed that, in patients with AF, aged ≥ 80 years, and deemed ineligible for oral anticoagulation at approved doses, edoxaban 15 mg od reduced the risk of stroke and systemic embolism by 66% compared with placebo, without a significant increase in the incidence of major bleeding [114]. In the frail subgroup of patients, ischemic events were reduced by 65%, with no increase in major bleeding events.

Motivated by these results, a further analysis was conducted. The efficacy and safety of very-low-dose of DOACs, namely, apixaban 2.5 mg od, edoxaban 15 mg od, rivaroxaban 10 mg od (patients without chronic kidney disease) or rivaroxaban < 10 mg od, and dabigatran 110/150 mg od, were compared with those of labeling-dosages of DOACs in high-risk elderly (age ≥ 80 years) AF patients at increased bleeding risk [115]. While the risk of ischemic events (stroke and systemic embolism) and major bleeding was comparable between the DOAC groups, the incidence of VTE increased by 3.8 times, death by 21%, and lower limb ischemic events by 57% with very low doses of DOACs compared with labeling-dosages. Therefore, the use of very-low-dose DOACs in elderly and frail patients instead of labeling-dosages for bleeding risk mitigation is not supported by current evidence.

## 9. Conclusions

We are witnessing a progressive increase in the geriatric population. With age, cardiovascular risk factors accumulate, leading to an increase in the prevalence of cardiovascular diseases. Among these, atrial fibrillation and venous thromboembolism are highly prevalent, both requiring anticoagulant treatment. Our review emphasizes that geriatric syndromes should not be feared nor used to justify refraining from anticoagulation. In patients at risk of falling, the risk–benefit balance is clearly in favor of anticoagulant treatment. Swallowing disorders and polypragmasy can be overcome and the consequences of forgetting to take a dose can be minimized by knowing the correct steps to follow. Furthermore, we provide data to help guide the choice of anticoagulant and dose. Thus, our work offers benchmarks with immediate application in current clinical practice.

## Figures and Tables

**Table 1:** Side effects of DOACs to consider in a patient that falls.

Drug	Side Effect	Frequency	Reference
Apixaban	Dizziness	Common	[26]
	Syncope	Uncommon	
	Hypotension	Common	
	Decreased general strength and energy (incl. fatigue and asthenia)	Common	
Edoxaban	Dizziness	Common	[27]
Rivaroxaban	Dizziness	Common	[28]
	Syncope	Uncommon	
	Hypotension	Common	
	Decreased general strength and energy (incl. fatigue and asthenia)	Common	

Common: frequency ≥ 1/100 to <1/10. Uncommon: frequency ≥ 1/1000 to <1/100.

**Table 2:** Administration of DOACs in patients with swallowing disorders or enteral tubes.

Drug	Solvent for Crushed Tablets	Reference
Apixaban	Orally: water, glucose 5%, apple juice or apple puree NGT: 60 mL water or glucose 5%	[26]
Edoxaban	Orally: water or apple puree NGT: water	[27]
Rivaroxaban	Orally: water or applesauce NGT: water or applesauce	[68]
Dabigatran	Crushing is not allowed.	[69]

**Table 3:** DOAC of choice in difficult combinations.

Drug	DOAC
Amiodarone	A, E
Dronedarone	A
Diltiazem	A, E, D
Verapamil	A, E
Carbamazepine	A, E
Phenytoin	A, E
Phenobarbital	A, E, D
Valproic acid	Contraindicated

A = apixaban, E = edoxaban, D = dabigatran.

**Table 4:** Recommended doses for DOACs in patients with AF or acute VTE.

		Standard Dose	Reduced Dose	Criteria for Dose Reduction
Factor Xa inhibitors				
APIXABAN	AF	5 mg bid	2.5 mg bid	If ≥2 of the following: Age ≥ 80 years; Weight ≤ 60 kg; SerCr ≥ 1.5 mg/dL. or CrCl 15–29 mL/min.
	VTE	10 mg bid, 7 days, then 5 mg bid	-	-
EDOXABAN	AF	60 mg od	30 mg od	If any of the following: Weight ≤ 60 kg; CrCl 15–50 mL/min; Concomitant therapy with strong P-gp inhibitor (ciclosporin, dronedarone, erythromycin, or ketoconazole).
	VTE	LMWH ≥ 5 days, then 60 mg od	LMWH ≥ 5 days, then 30 mg od	If any of the following: Weight ≤ 60 kg; CrCl 15–50 mL/min; Concomitant therapy with strong P-gp inhibitor (ciclosporin, dronedarone, erythromycin, or ketoconazole).
RIVAROXABAN	AF	20 mg	15 mg	CrCl 15–49 mL/min
	VTE	15 mg bid, 21 days, then 20 mg od	-	-
Factor IIa inhibitor				
DABIGATRAN	AF	150 mg bid	110 mg bid	Recommended, if any of the following: Age ≥ 80 years; Concomitant verapamil. Considered on an individual basis, if any of the following: Age 75–80 years; CrCl 30–50 mL/min; Gastritis, esophagitis, or gastro-esophageal reflux; Increased risk of bleeding.
	VTE	LMWH ≥ 5 days, then 150 mg bid	-	-

## Data Availability

Data sharing is not applicable to this article.
